# *Helicobacter pylori* infection and its impact on psoriasis: a systematic review and meta-analysis

**DOI:** 10.3389/fmed.2024.1500670

**Published:** 2024-12-06

**Authors:** Yijiao Yan, Wenhui Deng, Chengzhi Shi, Jiaxin Xie, Daoshun Sui

**Affiliations:** ^1^The First Clinical Medical School of Guangzhou University of Chinese Medicine, Guangzhou, China; ^2^First Affiliated Hospital, Guangzhou University of Traditional Chinese Medicine, Guangzhou, China

**Keywords:** psoriasis, *Helicobacter pylori*, meta-analysis, immunology, PROSPERO

## Abstract

**Introduction:**

Psoriasis is a chronic skin condition characterized by immune-mediated inflammation. Recent research suggests a possible interaction between *Helicobacter pylori* infection and the immunopathogenesis of psoriasis. However, over the past 5 years, no significant new evidence has clarified the relationship between *H. pylori* and skin diseases. This study aimed to determine the relationship between *H. pylori* infection and psoriasis through a systematic review and meta-analysis.

**Methods:**

We searched for articles published in databases including PubMed, Embase, the China National Knowledge Infrastructure, and Web of Science up to January 1, 2024. Statistical analyses were conducted using Review Manager 5.3 and Stata 12.0 software.

**Results:**

Our search yielded 271 papers. After rigorous screening by multiple reviewers, 15 studies involving 2,427 individuals were included. The odds ratio for *H. pylori* infection was significantly higher in the psoriasis group than in the control group (odds ratio = 1.94, 95% confidence interval: 1.40–2.68, *p* < 0.0001). Subgroup analysis revealed no significant differences in *H. pylori* infection rates between Asia and Europe. The type of study also did not significantly affect infection rates. The enzyme-linked immunosorbent assay detected *H. pylori* infection at a significantly higher rate than the breath test. Furthermore, the prevalence of *H. pylori* infection differed significantly between patients with moderate-to-severe psoriasis and those with mild psoriasis.

**Conclusion:**

Our findings suggest a relationship between psoriasis and *H. pylori* infection, with variations observed based on geography, testing methods, and disease severity. These findings hold significant potential for guiding clinical practice.

**Systematic review registration:**

http://www.crd.york.ac.uk/, identifier CRD42022359427.

## 1 Introduction

Psoriasis is a chronic skin condition characterized by immune-mediated inflammation (1). The severity of the disease can range from a few localized red, scaly patches to widespread involvement, affecting almost the entire body surface (2). Studies have shown that the prevalence of psoriasis is influenced by both age and geographic location (3, 4), with higher rates observed in countries farther from the equator. Reported prevalence rates in adults range from 0.91% in the United States to 8.5% in Norway. In the United States, the incidence rate among the pediatric population is estimated at 40.8 cases per 100,000 person-years. Among adults, incidence rates vary significantly, from 78.9 per 100,000 person-years in the United States to 230 per 100,000 person-years in Italy (5).

The precise etiology of psoriasis is not yet fully understood; however, genetic, immunological, and environmental factors are believed to contribute to its pathogenesis (2, 6). Various microorganisms have been increasingly implicated in the onset and exacerbation of psoriasis (7). For mild cases, topical medications and phototherapy are effective treatment options. Conversely, severe cases may require systemic therapies, such as biologics and small-molecule targeted drugs (8), which are tailored to individual needs. *Helicobacter pylori*, a gram-negative bacterium commonly found in the stomach, has been classified by the World Health Organization as a Group 1 carcinogen due to its association with gastric inflammation, ulceration, and potential malignant transformations (9, 10). Current research suggests a potential interaction between *H. pylori* infection and the immunopathogenesis of psoriasis (8, 11), with evidence indicating a possible link between the bacterium and disease severity (12). The eradication of *H. pylori* has been shown to improve treatment outcomes in patients with psoriasis. Additionally, microbial heat shock proteins (HSPs) are thought to play a role in autoimmune disease pathogenesis due to their high sequence homology with human HSPs (13). This principle extends beyond psoriasis, as *H. pylori* eradication has also proven effective in alleviating symptoms of chronic urticarial (14).

Recent research suggests that *H. pylori* may contribute to the development of several skin conditions, with the strongest evidence linking it to chronic urticarial (15) and immune thrombocytopenic purpura (16). Numerous studies in recent years have confirmed these associations (17), and some research groups have used meta-analytic techniques to systematically review the available literature (18, 19). However, in the past 5 years, no significant new evidence has emerged to deepen our understanding of the link between *H. pylori* and skin diseases. Although meta-analyses have been conducted on data available up to 2019, a substantial gap remains in the literature for the subsequent period. This study aimed to address this gap by re-examining all available research on the relationship between *H. pylori* infection and psoriasis up to January 1, 2024. By supplementing existing data, we hope to provide more robust evidence to guide clinical practice. The findings of this study offer compelling evidence of an association between *H. pylori* infection and psoriasis (20).

## 2 Materials and methods

### 2.1 Study design

This study followed the Preferred Reporting Items for Systematic Reviews and Meta-analyses Protocols (PRISMA-P) 2015 guidelines (21). A meta-analytic approach was used to ensure the highest standard of clinical evidence. Given the scarcity of recent data, our team supplemented the dataset with new findings. We utilized RevMan 5.3 and Stata software to analyze database search results and identify studies eligible for inclusion. Following a rigorous data-cleaning process, we identified 15 studies that met the inclusion criteria. This study design is recognized for its high evidentiary value, supporting the principles of evidence-based medicine. It exclusively utilizes data from established databases up to the present, acknowledging the inherent limitations of data availability. Despite these constraints, we made efforts to reduce potential biases in our data analysis.

### 2.2 Search strategy

We systematically searched PubMed, Web of Science, Embase, and the China National Knowledge Infrastructure databases to identify all relevant studies on the relationship between *H. pylori* infection and psoriasis published up to January 1, 2024. Our search criteria included all languages and article types, with no exclusions. Table 1 outlines the comprehensive search strategy.

**Table 1:** Search strategy.

#1 Psoriasis
#2 Psoriases
#3 Palmoplantaris pustulosis
#4 #1 OR #2 OR #3
#5 *Helicobacter pylori*
#6 *H. pylori*
#7 #5 OR #6
#8 #4 AND #7

This table shows the detailed search terms used in the major database searches.

### 2.3 Inclusion criteria

Two independent reviewers, YY and WD, conducted an initial screening of the retrieved search results. Studies were considered eligible based on the following inclusion criteria:

1. Studies employing a cohort, cross-sectional, or case-control design to investigate the relationship between psoriasis and *H. pylori* infection.
2. Studies comparing at least two distinct groups: (a) individuals with a confirmed diagnosis of psoriasis via clinical assessment or histopathology, and (b) control participants without psoriasis from hospital or community settings.
3. Studies for which the full text was available for review.
4. Studies reporting the prevalence of *H. pylori* infection in both psoriasis and control groups.
5. Studies in which *H. pylori* infection was diagnosed through histological examination, IgG enzyme-linked immunosorbent assay (ELISA), urea breath testing, or stool antigen testing.

### 2.4 Exclusion criteria

The study selection process considered the following exclusion criteria:

1. Studies published as meeting abstracts, case reports, editorial comments, correspondence letters, or review articles.
2. Studies involving participants with pre-existing conditions, such as cardiovascular or renal diseases, as well as those who used medications—including antibiotics, proton pump inhibitors, antacids, or glucocorticoids—within 2 weeks prior to study initiation.
3. Studies with incomplete data; when duplicate reports were identified or when studies reported results from overlapping populations, the most recent and comprehensive study was included.

### 2.5 Data extraction

Two reviewers, YY and WD, independently extracted data using pre-defined, standardized abstraction forms. Discrepancies between the reviewers were resolved through consultation with a third reviewer (CS), ensuring consistency in the study selection and data extraction process. Data extracted from each study included the lead author’s name, year of publication, study setting, design, baseline characteristics of participants, Psoriasis Area and Severity Index (PASI) scores, and *H. pylori* infection testing results. When essential details were missing from the original articles, corresponding authors were contacted for clarification.

### 2.6 Quality assessment

The Newcastle–Ottawa Scale (22) was used to assess the quality of the included studies, with scores ranging from 0 to 9. A higher score indicates better study quality, classified as follows: 0–3 for poor, 4–6 for fair, and 7–9 for good quality. Quality assessment was independently conducted by two reviewers, YY and WD, with any disagreements resolved through discussion to reach a consensus.

### 2.7 Data analysis

The meta-analysis was performed using Review Manager 5.3 and Stata 12.0. We determined the prevalence of *H. pylori* infection in both psoriasis and control groups, presenting the results as odds ratios (ORs) with their respective 95% confidence intervals (CIs). A *p*-value of <0.05 was considered statistically significant. Heterogeneity among the studies was measured using the chi-square test and evaluated with inconsistency statistics, classified as follows: 0–25% for homogeneity, 25–50% for low heterogeneity, 50–75% for moderate heterogeneity, and >75% for high heterogeneity (23). Subgroup analyses were conducted to explore variables such as geography, testing methodology, study design, and psoriasis severity.

## 3 Results

The results, presented in both graphical and narrative formats, revealed a strong correlation between *H. pylori* infection and psoriasis. Subgroup analyses indicated that this correlation might be influenced by the *H. pylori* detection method and psoriasis severity, with testing method showing a lower impact. These findings indicate that bactericidal therapy may be necessary in the clinical management of *H. pylori*-positive patients with psoriasis, potentially as part of a combination therapy for those with moderate-to-severe psoriasis. Although our dataset is robust, it is not exhaustive and may contain some bias. To mitigate this, we endeavored to include as much high-quality data as possible. Some studies were excluded because they did not meet the inclusion criteria, such as comment articles, although they were related to psoriasis and *H. pylori* infection (24).

### 3.1 Literature search and study characteristics

**Figure 1:**
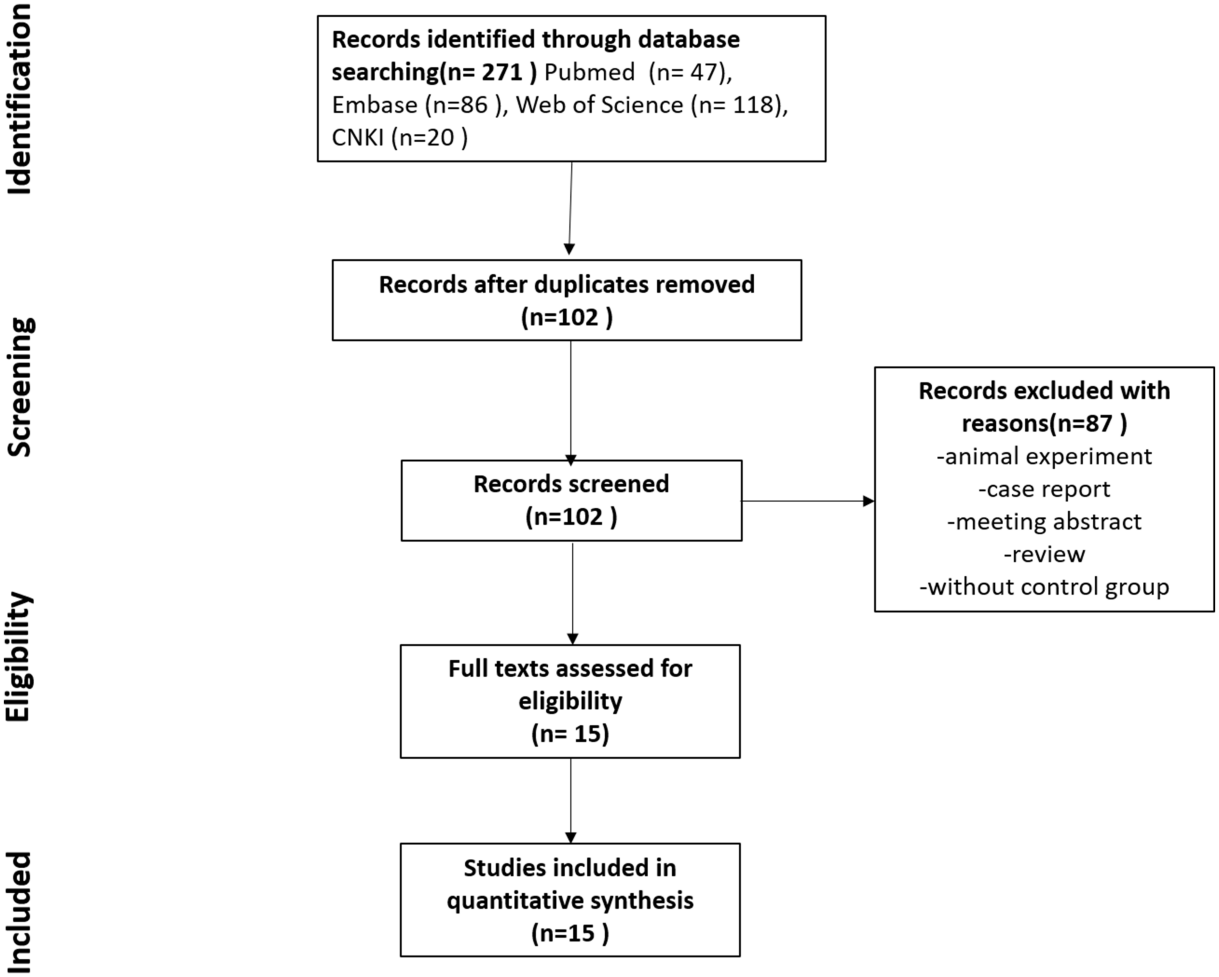
Study selection flowchart. This figure shows the complete process of literature screening. A search of four databases yielded 271 articles, with 102 articles remaining after deduplication. The titles, abstracts, and keywords of these 102 articles were carefully reviewed, and 15 articles meeting the inclusion criteria and with full texts available were included in this study.

We conducted a comprehensive search of major databases, yielding 271 articles. After deduplication, which removed 169 duplicates, 102 unique papers remained. Using the aforementioned inclusion criteria, we meticulously screened the titles, abstracts, and full texts, ultimately selecting 15 papers for inclusion in this meta-analysis. Figure 1 illustrates the selection process, and Table 2 presents all included studies with detailed information. We assessed the quality of each article using the Newcastle–Ottawa Scale score; all included articles scored above 7, except for one study by Spanish authors in 2000. Specific scores are detailed in Table 2.

**Table 2:** Included studies and data extraction.

Study	Country	Study design	Total cases	Women	Mean age	Mean PASI	Outcomes	NOS score
Xiao-Hong and Yun-Sheng (40)	China	Case-control	146 (86/60)	35.6	16.5–70.5	15.62 ± 8.19	Positive urea breath test	7
Azizzadeh et al. (41)	Iran	Case-control	122 (61/61)	54	33.3	6.6 ± 3.1	Positive *H. pylori* IgG ELISA test	7
Mesquita et al. (26)	Brazil	Cohort study	147 (126/21)	57.9	50.48	PASI <5 = 21	Positive *H. pylori* IgG ELISA test	8
						PASI 5–10 = 40		
						PASI >10 = 65		
Aihemaiti (42)	China	Case-control	200 (100/100)	32	42.69 ± 13.57	PASI <5 = 18	Positive *H. pylori* IgG ELISA test	7
						PASI 5–10 = 43		
						PASI >10 = 39		
Campanati et al. (27)	Italy	Cohort study	360 (210/150)	48.1	49.75	14.56 ± 4.35	Positive urea breath test	9
Onsun et al. (17)	Turkey	Cohort study	450 (300/150)	49	41.65	3.94 ± 4.99	Positive stool antigen test	7
Daudén et al. (43)	Spain	Case-control	145 (84/61)	NA	NA	NA	Positive urea breath test	4
Fabrizi et al. (44)	Italy	Case-control	49 (20/29)	44.9	5–19	NA	Positive urea breath test	6
Türkmen et al. (45)	Turkey	Cross-sectional study	113 (56/57)	42.9	38.4	5.89	Positive urea breath test	7
Zhelezova et al. (46)	Bulgaria	Cross-sectional study	49 (25/24)	32	52.2	NA	Positive *H. pylori* IgG ELISA test	7
Qayoom and Ahmad (47)	India	Cross-sectional study	100 (50/50)	44	5–60	NA	Positive *H. pylori* IgG ELISA test	7
Minghua and Hengjin (48)	China	Case-control	97 (62/35)	36.10	17–66	NA	Positive urea breath test	8
Xie (49)	China	Case-control	144 (72/72)	41.0	35.6 ± 7.18	17.42 ± 3.43	Positive urea breath test	7
Huang et al. (50)	China	Cohort study	191 (103/88)	40.78	9–74	NA	Positive *H. pylori* IgG ELISA test	7
Chen et al. (51)	China	Cross-sectional study	94 (64/30)	NA	NA	NA	Positive *H. pylori* IgG ELISA test	7

This table provides detailed data on all included studies, including geographic area, sample size, sex ratio, mean age, mean PASI score, *H. pylori* testing methods, and NOS scores. NA, not available; NOS, Newcastle–Ottawa Scale.

### 3.2 *Helicobacter pylori* infection rates

**Figure 2:**
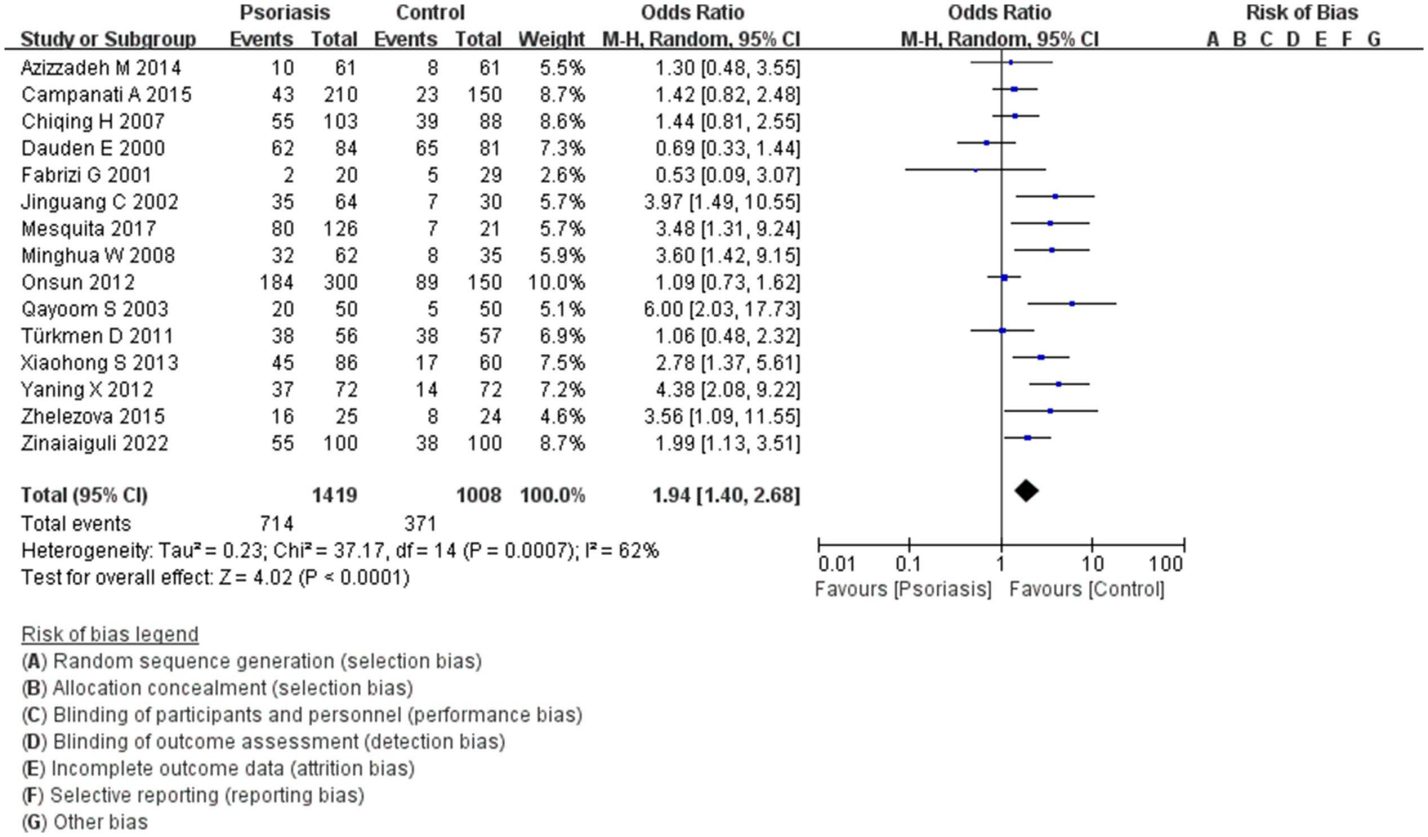
*H. pylori* infection in patients with or without psoriasis. This figure shows the correlation between the prevalence of *H. pylori* infection and psoriasis, analyzed using RevMan software. The final statistical results indicated a significant difference in the rate of *H. pylori* infection in patients with psoriasis compared to the general population.

This study included 1,419 patients with psoriasis and 1,008 control participants. The prevalence of *H. pylori* infection was 50.3% in the psoriasis group and 36.8% in the control group. As shown in Figure 2, the pooled OR was 1.94, with a 95% CI of 1.40–2.68. This result was statistically significant (*p* < 0.0001).

### 3.3 Subgroup analysis

**Figure 3:**
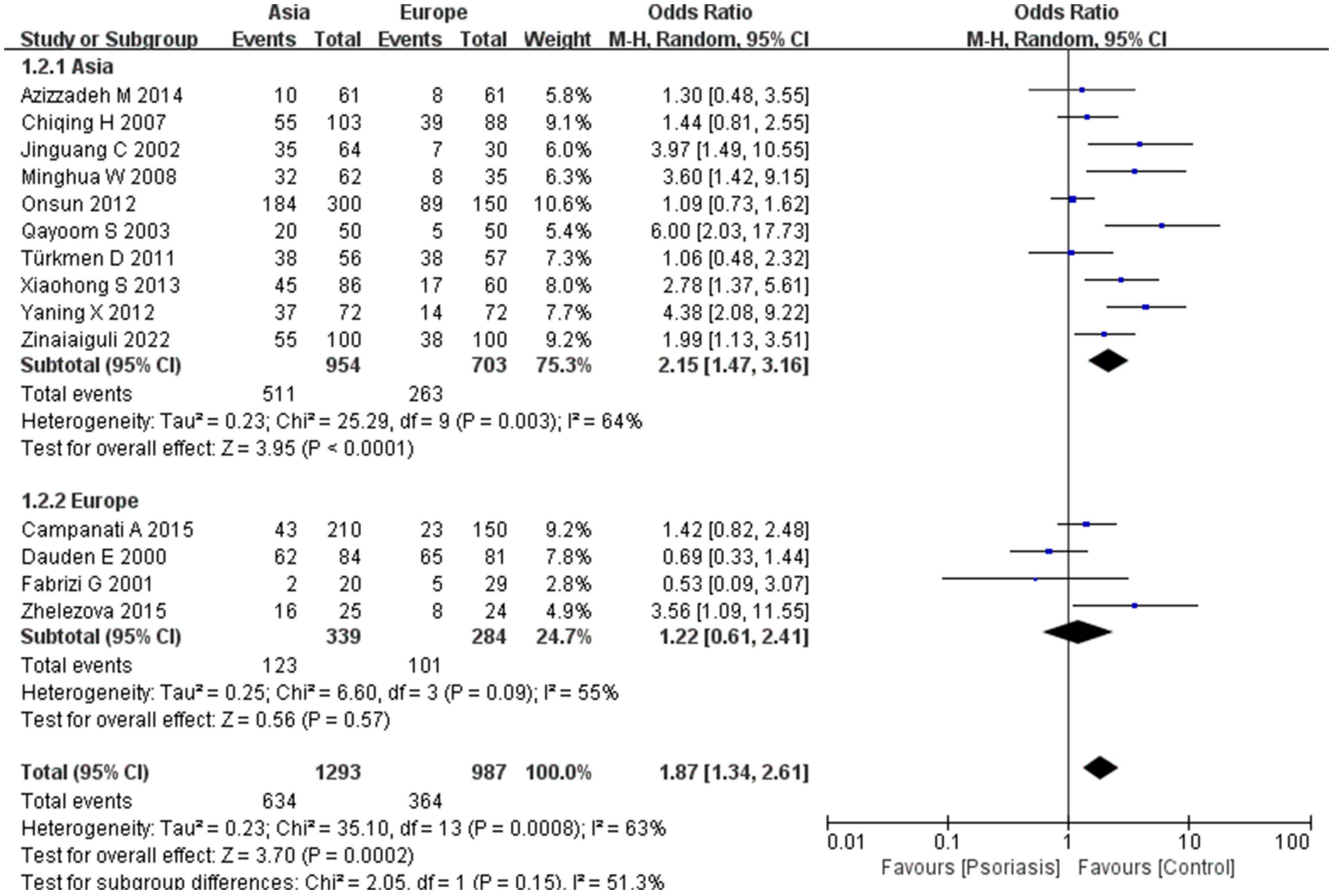
Sub analysis based on geography: Asia and Europe.

**Figure 4:**
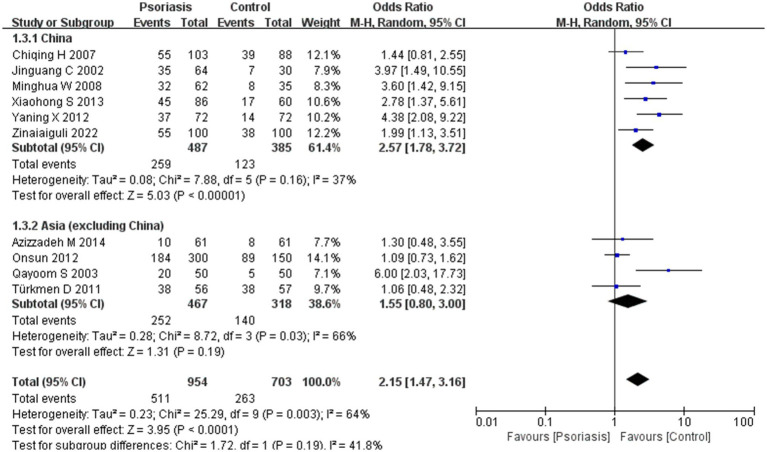
Sub analysis based on geography: China and other Asian countries.

**Figure 5:**
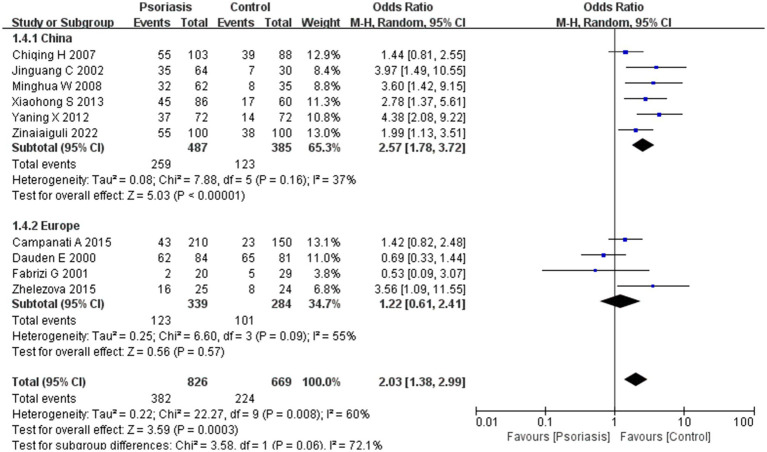
Sub analysis based on geography: China and Europe.

**Figure 6:**
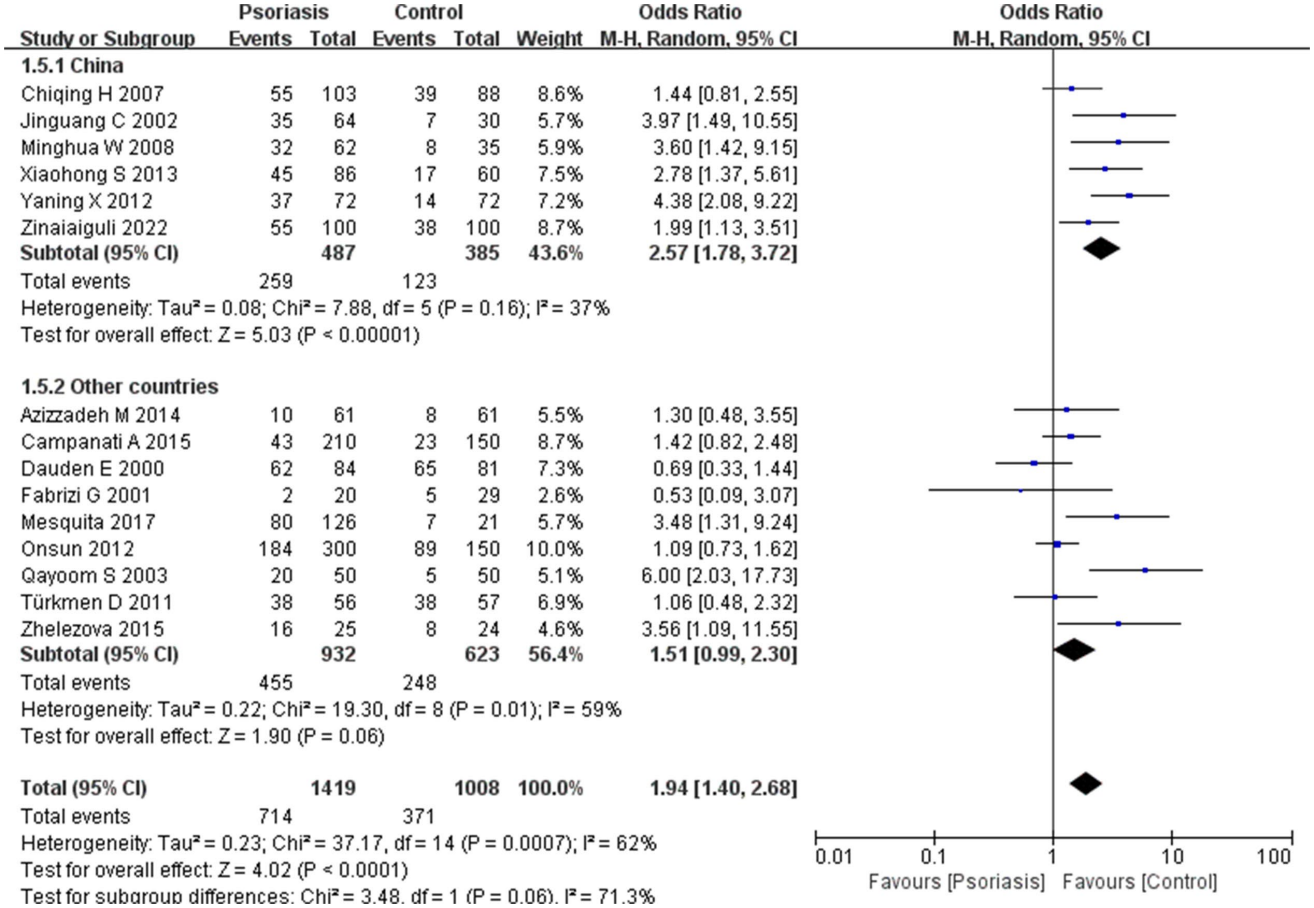
Sub analysis based on geography: China and other countries. These figures show the first subgroup analysis conducted using RevMan software to estimate the variability of *H. pylori* infection rates across different geographical areas (Asia and Europe). The results indicated no significant difference in infection rates between Asia and Europe. When data from China were analyzed separately, the results showed that the prevalence of *H. pylori* infection in psoriasis patients in China was not significantly different from that in other countries.

**Figure 7:**
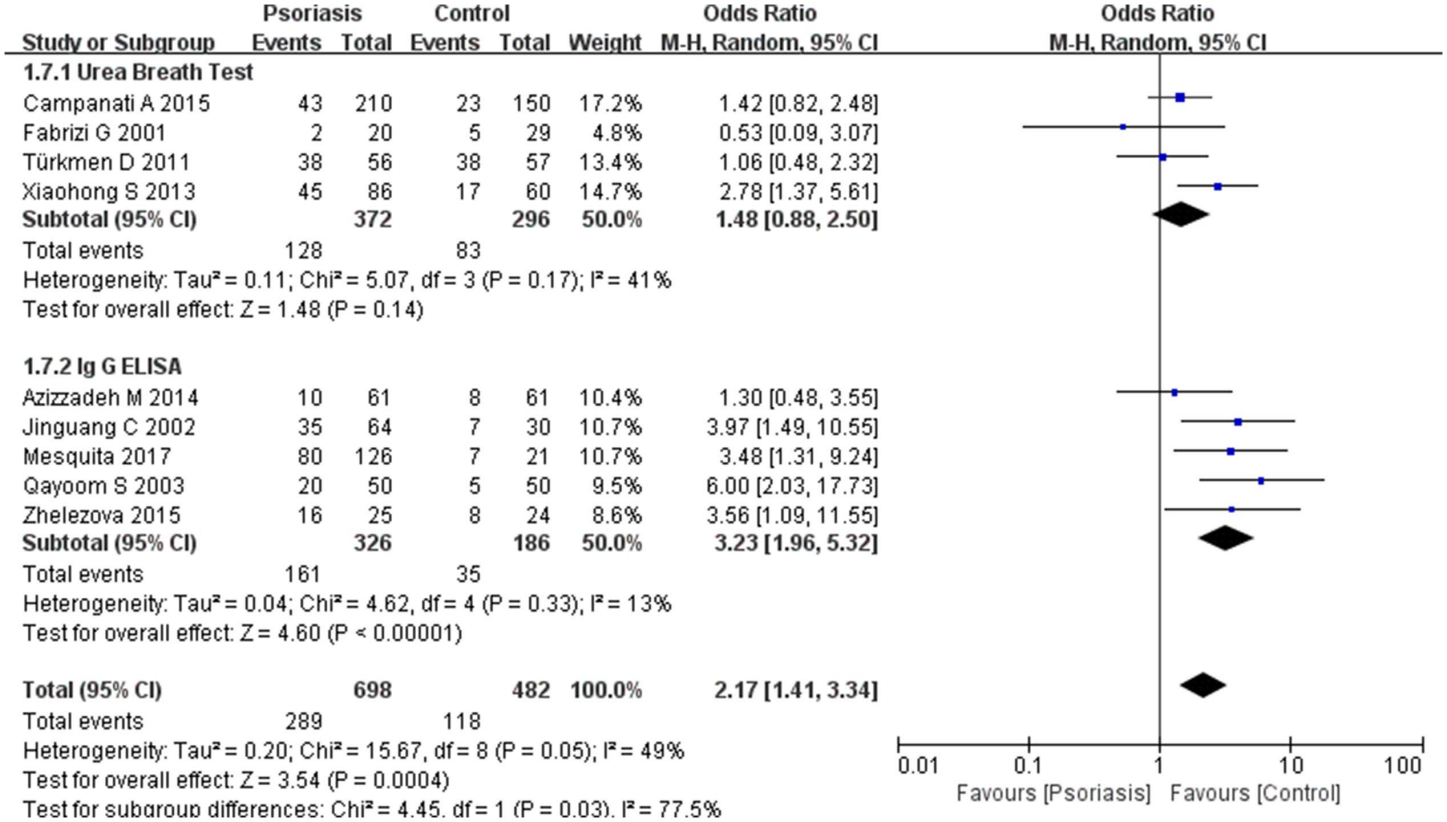
Sub analysis based on *H. pylori* detection methods. This figure shows the second subgroup analysis performed using RevMan software to estimate the variability of *H. pylori* infection rates across different assays (urea breath test vs. ELISA). The results indicated that ELISA yielded a significantly higher *H. pylori* positivity rate than the urea breath test.

**Figure 8:**
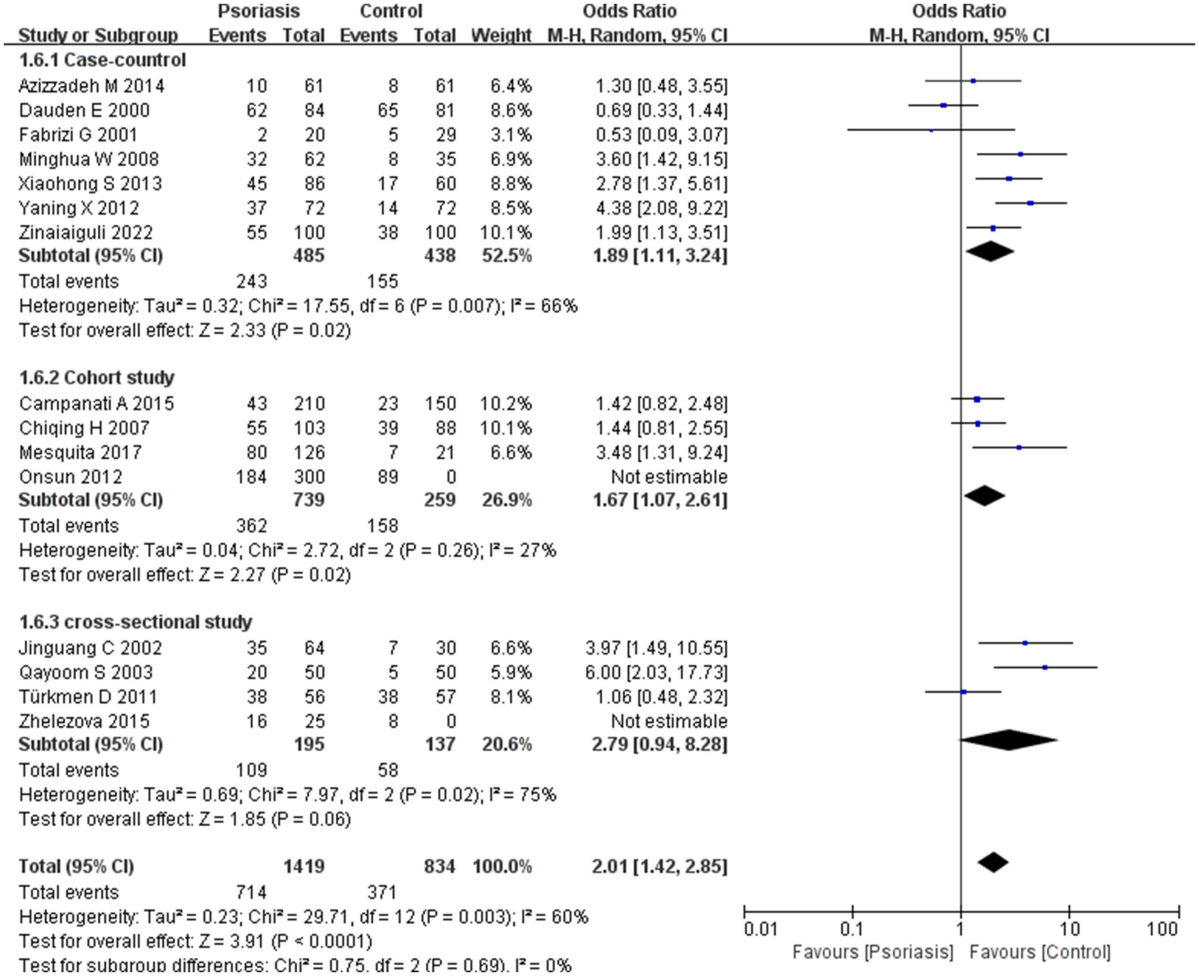
Sub analysis based on study design. This figure shows the results of subgroup analyses across different study types, indicating that study type is not an influential factor in the prevalence of *H. pylori* infection, with no variability observed between study types.

**Figure 9:**
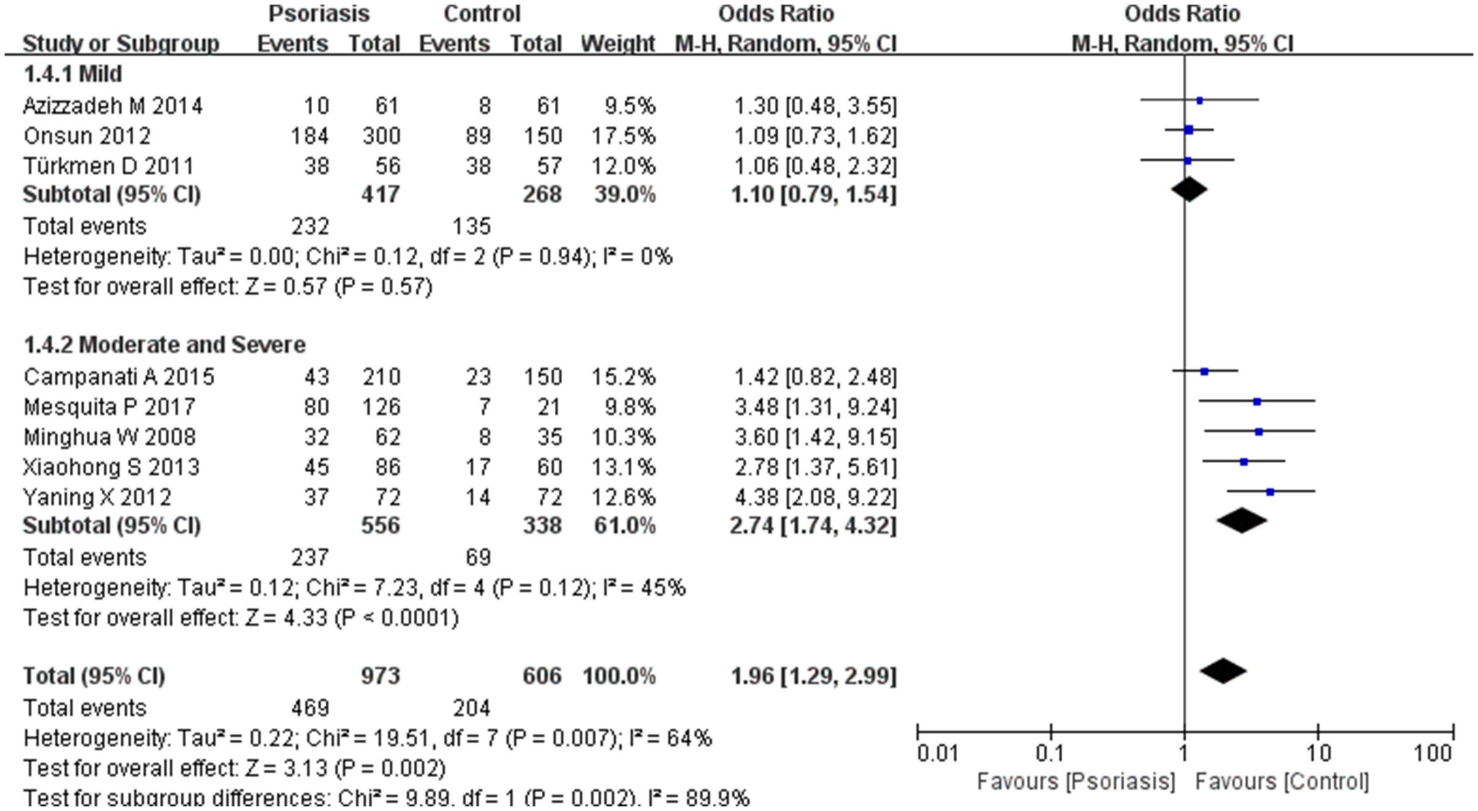
Sub analysis based on psoriasis severity. This figure shows the third subgroup analysis performed using RevMan software to estimate the variability of *H. pylori* infection rates across different severity levels of psoriasis. The results indicated that patients with moderately severe psoriasis had a significantly higher rate of *H. pylori* infection than those with mild psoriasis.

Our meta-analysis was stratified by several factors, including geographic location (Asia or Europe; Figure 3, Asia or China; Figure 4, China or Europe; Figure 5, and China or other countries; Figure 6), *H. pylori* detection method (Figure 7), study design (Figure 8), and psoriasis severity, as measured using the PASI score. The PASI score categorizes psoriasis as mild (mean PASI ≤10), moderate (mean PASI ≥10 to <20), or severe (mean PASI ≥20) (Figure 9). Geographic subgroup analyses showed no significant differences in the prevalence of *H. pylori* infection in psoriasis patients when comparing Asia with Europe, China with other Asian countries, or China with Europe and other countries. Among these, the Asia vs. Europe subgroup analysis showed an OR of 1.87 with a 95% CI of 0.61–2.41, *p*-value of 0.15, and *I*^2^ of 51.3%. The China vs. other Asian countries subgroup analysis showed an OR of 2.15 with a 95% CI of 1.47–3.16, *p*-value of 0.19, and *I*^2^ of 41.8%. The China vs. Europe subgroup analysis showed an OR of 1.22 with a 95% CI of 1.38–2.99, *p*-value of 0.06, and *I*^2^ of 72.1%. Lastly, the China vs. other countries subgroup analysis yielded an OR of 1.94 with a 95% CI of 0.99–2.30, *p*-value of 0.06, and *I*^2^ of 71.3%.

Subgroup analyses comparing different *H. pylori* detection methods revealed a statistically significant difference in positivity rates between the urea breath test and ELISA, with ELISA detecting a higher rate of *H. pylori* infection. The assay subgroup analysis showed an OR of 2.17 with a 95% CI of 1.41–3.34, *p*-value of 0.03, and *I*^2^ of 77.5%.

The included studies were categorized into case-control, cohort, and cross-sectional studies based on study design. Subgroup analysis suggested no significant difference in *H. pylori* infection prevalence between study types, with an OR of 2.01, 95% CI of 1.42–2.85, *p*-value of 0.69, and *I*^2^ of 0%.

Subgroup analyses suggested a correlation between psoriasis severity and *H. pylori* infection prevalence, which was significantly higher in patients with moderately severe psoriasis than in those with milder forms. The psoriasis severity subgroup analysis showed an OR of 1.96, 95% CI of 1.29–2.99, *p*-value of 0.002, and *I*^2^ of 89.9%.

### 3.4 Sensitivity analysis and publication bias

After excluding studies individually, we found no significant difference in the overall risk of *H. pylori* infection. Additionally, we did not observe significant publication bias, as assessed using the Begg (*p* = 0.198) and Egger (*p* = 0.114) tests.

## 4 Discussion

We conducted a meta-analysis to investigate the relationship between psoriasis and *H. pylori* infection by systematically searching several major databases and performing statistical analyses using RevMan and Stata software. Our findings suggest a link between *H. pylori* infection and psoriasis, influenced by the *H. pylori* detection method and psoriasis severity (25, 26). A previous study highlighted the utility of the ^13^C-urea breath test and recommended *H. pylori* eradication therapy before initiating psoriasis treatment to reduce inflammation (27). Although this study’s dataset is substantial, we recognize that future research should include larger, multicenter clinical trials to validate the observed correlation and investigate underlying mechanisms. Additionally, future studies should assess whether patients with psoriasis who receive *H. pylori* eradication therapy experience significant improvements. Our findings provide preliminary evidence that may help guide the development of combination therapies for clinical management of patients with both psoriasis and *H. pylori* infection. We also remain open to the emergence of new high-quality clinical studies that may offer further insights and complement our data.

To date, two meta-analyses (18, 19) have investigated the link between psoriasis and *H. pylori* infection, both finding that patients with psoriasis have a higher risk of *H. pylori* infection. Our study expands on previous research by incorporating additional data and conducting subgroup analyses. These analyses revealed no significant differences in infection rates between the Asian and European regions and that differences in study type did not affect infection rates. However, the ELISA test showed a higher positive rate compared to the breath test. Based on PASI scores, patients with moderate-to-severe psoriasis had a higher prevalence of *H. pylori* infection.

In our study on the link between *H. pylori* infection and psoriasis, the results revealed a statistically significant correlation, with a *p*-value of <0.001, OR of 1.94, and 95% CI of 1.40–2.68. The chi-square value was 62%, indicating moderate heterogeneity among the studies, possibly reflecting the variable quality of the included literature. These findings suggest a potential link between *H. pylori* infection and the development of psoriasis, supported by the high evidentiary value of meta-analysis in evidence-based medicine. A previous study associated the pathogenesis of psoriasis with immune system involvement (28). Insights into the role of immune function in psoriasis, particularly the interaction between the innate and adaptive immune systems, have been critical for managing this multifaceted disease, which impacts patients beyond the skin level (29, 30). Although current research has identified links between psoriasis and gastrointestinal immune-related disorders such as Crohn’s disease, there are insufficient large, multicenter clinical trials to definitively link psoriasis with *H. pylori* infection. Furthermore, the underlying mechanism for this relationship remains unclear. If such a mechanism is identified, it may be possible to incorporate *H. pylori* eradication therapy into psoriasis treatment regimens. Further research is needed to assess the potential efficacy of this therapeutic approach.

In the initial subgroup analysis, we compared the *H. pylori* infection rate between Asian and European patients with psoriasis and found no significant geographic differences. Further subgroup analyses comparing China with other Asian countries, China with Europe, and China with other countries also indicated that geography was not a factor influencing *H. pylori* infection rates in patients with psoriasis. Earlier studies have shown that *H. pylori* infection rates in China are higher than the average rate in developed countries (31), potentially due to economic conditions and dining habits. Notably, lower rates of *H. pylori* infection have been reported in young people, in high-income countries or countries with high universal health insurance coverage, and in retrospective studies (32). However, we found no evidence of geographic differences affecting *H. pylori* infection rates, especially in patients with psoriasis. In China, there was a high rate of familial *H. pylori* infection within households, with exposure to infected family members being the most common mode of transmission. Our results suggest that there is no significant difference in the prevalence of *H. pylori* infection in patients with psoriasis between Asia and Europe, suggesting that geographic location is not a primary factor in infection rates among these patients.

In a subsequent subgroup analysis, we found a significant difference between the urea breath test and ELISA for detecting *H. pylori* (33). The urea breath test is currently preferred over the IgG ELISA as the primary diagnostic method. However, these two methods differ in sensitivity and specificity, which should be considered in clinical practice (34).

Our third subgroup analysis, which excluded heterogeneity due to study type, showed that the prevalence of *H. pylori* infection did not correlate with study type.

Our fourth subgroup analysis focused on the relationship between *H. pylori* infection prevalence and psoriasis severity. We observed no significant difference in *H. pylori* infection rates between the mild psoriasis and control groups. Conversely, a significant difference was found in the moderate-to-severe psoriasis subgroup, with higher prevalence observed in this group. This suggests that *H. pylori* infection is more common in patients with moderate-to-severe psoriasis than in those without psoriasis, potentially linking infection rate to psoriasis severity, particularly in more severe cases. This correlation may be attributed to immune mechanisms activated by the infection. Considering the varying severity of psoriasis, future studies should re-evaluate patients after *H. pylori* treatment to assess outcomes. This would involve determining whether changes in PASI scores are statistically significant, which may help guide the clinical use of adjunctive treatments for psoriasis.

In addition to genetic predisposition, several nongenetic factors influence the onset and recurrence of psoriasis. These factors include infections, imbalances in skin and gut microbiota, disruptions in lipid metabolism, irregularities in sex hormones, and mental health issues (35, 36). Studies have also suggested associations between *H. pylori* infection and dermatological conditions (such as psoriasis and rosacea) and open-angle glaucoma (7, 37). The precise mechanisms underlying these associations require further investigation (38).

Interestingly, the incidence of psoriasis is somewhat lower in survivors of gastric cancer than in the general population. Our findings indicate that the risk of developing psoriasis may be reduced by *H. pylori* eradication, a known source of systemic inflammation, through subtotal gastrectomy in individuals who have undergone treatment for gastric cancer.

*H. pylori* infection has long been associated with various diseases, including gastric ulcers and gastric cancer; however, universal screening and eradication treatments seem to contribute to the development of resistance on a larger scale (39), creating a therapeutic paradox. Whether screening and eradicating *H. pylori* in patients with psoriasis improves patient prognosis requires further investigation. While the results of this study confirm the correlation, limitations remain in establishing causality.

This study has some limitations. First, our study included literature from only four databases in Chinese and English, which may limit its applicability in a global context. Some of these studies had small sample sizes, introducing a potential for bias. Second, our study focused on the relationship between *H. pylori* infection and psoriasis, with most included studies concluding that *H. pylori* prevalence is higher in psoriasis patients than in those without psoriasis. However, there are no clinical trials confirming whether *H. pylori* eradication therapy leads to an improvement in psoriasis skin lesions or whether populations treated for *H. pylori* infection have a lower incidence of psoriasis relative to epidemiologic data. These limitations highlight areas for further research.

## 5 Conclusion

In this study, we conducted a systematic review and meta-analysis of existing literature on the relationship between *H. pylori* infection and psoriasis. Our analysis revealed a significant correlation between the two conditions, providing valuable insights for future etiological research on psoriasis. The results showed no significant difference in the prevalence of *H. pylori* infection between studies from China and other countries, and *H. pylori* detection using ELISA had a higher positive rate compared to breath testing. Differences in study type did not affect infection rates. Subgroup analyses also indicated that individuals infected with *H. pylori* may have higher PASI scores. However, it is important to acknowledge the limitations of our study, which was restricted to a few online databases and may have missed relevant physical literature. Additionally, the impact of *H. pylori* treatment on psoriasis progression requires further investigation. Our findings confirm a link between *H. pylori* infection and psoriasis, which could serve as a guide for future clinical management of psoriasis, including the potential use of antimicrobial therapies.

## Data Availability

The original contributions presented in the study are included in the article/Supplementary material, further inquiries can be directed to the corresponding author.

## References

[1] Liu S He M Jiang J Duan X Chai B Zhang J . Triggers for the onset and recurrence of psoriasis: a review and update. Cell Commun Signal. (2024) 22:108. doi: 10.1186/s12964-023-01381-0, PMID: 38347543 PMC10860266

[2] Ayala-Fontanez N Soler DC Mccormick TS. Current knowledge on psoriasis and autoimmune diseases. Psoriasis. (2016) 6:7–32. doi: 10.2147/PTT.S64950, PMID: 29387591 PMC5683130

[3] Griffiths CEM van der Walt JM Ashcroft DM Flohr C Naldi L Nijsten T . The global state of psoriasis disease epidemiology: a workshop report. Br J Dermatol. (2017) 177:e4–7. doi: 10.1111/bjd.15610, PMID: 28555722 PMC5600082

[4] Eltaweel A. Chapter 1—epidemiology, aetiology and symptomatology. J Eur Acad Dermatol Venereol. (2016) 30:83. doi: 10.1111/jdv.2_13848 27558687

[5] Parisi R Symmons DPM Griffiths CEM Ashcroft DM. Global epidemiology of psoriasis: a systematic review of incidence and prevalence. J Invest Dermatol. (2013) 133:377–85. doi: 10.1038/jid.2012.339 23014338

[6] Orsmond A Bereza-Malcolm L Lynch T March L Xue M. Skin barrier dysregulation in psoriasis. Int J Mol Sci. (2021) 22:10841. doi: 10.3390/ijms221910841, PMID: 34639182 PMC8509518

[7] Teng Y Xie W Tao X Liu N Yu Y Huang Y . Infection-provoked psoriasis: induced or aggravated (review). Exp Ther Med. (2021) 21:567. doi: 10.3892/etm.2021.9999, PMID: 33850539 PMC8027725

[8] Wedi B Kapp A. *Helicobacter pylori* infection in skin diseases. Am J Clin Dermatol. (2002) 3:273–82. doi: 10.2165/00128071-200203040-00005 12010072

[9] Malfertheiner P Camargo MC El-Omar E Liou JM Peek R Schulz C . *Helicobacter pylori* infection. Nat Rev Dis Primers. (2023) 9:19. doi: 10.1038/s41572-023-00431-8, PMID: 37081005 PMC11558793

[10] Salvatori S Marafini I Laudisi F Monteleone G Stolfi C. *Helicobacter pylori* and gastric cancer: pathogenetic mechanisms. Int J Mol Sci. (2023) 24:2895. doi: 10.3390/ijms24032895, PMID: 36769214 PMC9917787

[11] Tuzun Y Keskin S Kote E. The role of *Helicobacter pylori* infection in skin diseases: facts and controversies. Clin Dermatol. (2010) 28:478–82. doi: 10.1016/j.clindermatol.2010.03.002, PMID: 20797505

[12] Maev IV Andreev DN Kucheryavyi YA. *Helicobacter pylori* infection and extragastroduodenal diseases. Ter Arkhiv. (2015) 87:103–10. doi: 10.17116/terarkh2015878103-110, PMID: 28635878

[13] Magen E Delgado JS. *Helicobacter pylori* and skin autoimmune diseases. World J Gastroenterol. (2014) 20:1510–6. doi: 10.3748/wjg.v20.i6.1510, PMID: 24587626 PMC3925859

[14] Sakurane M Shiotani A Furukawa F. Therapeutic effects of antibacterial treatment for intractable skin diseases in *Helicobacter pylori*-positive Japanese patients. J Dermatol. (2002) 29:23–7. doi: 10.1111/j.1346-8138.2002.tb00160.x, PMID: 11837570

[15] Shiotani A Okada K Yanaoka K Itoh H Nishioka S Sakurane M . Beneficial effect of *Helicobacter pylori* eradication in dermatologic diseases. Helicobacter. (2001) 6:60–5. doi: 10.1046/j.1523-5378.2001.00009.x, PMID: 11328367

[16] Hernando-Harder AC Booken N Goerdt S Singer MV Harder H. *Helicobacter pylori* infection and dermatologic diseases. Eur J Dermatol. (2009) 19:431–44. doi: 10.1684/ejd.2009.0739 19527988

[17] Onsun N Arda Ulusal H Su O Beycan I Biyik Ozkaya D Senocak M. Impact of *Helicobacter pylori* infection on severity of psoriasis and response to treatment. Eur J Dermatol. (2012) 22:117–20. doi: 10.1684/ejd.2011.1579, PMID: 22063790

[18] Yu M Zhang R Ni P Chen S Duan G. *Helicobacter pylori* infection and psoriasis: a systematic review and meta-analysis. Medicina. (2019) 55:645. doi: 10.3390/medicina55100645, PMID: 31561576 PMC6843633

[19] Yong W Upala S Sanguankeo A. Association between psoriasis and *Helicobacter pylori* infection: a systematic review and meta-analysis. Indian J Dermatol. (2018) 63:193–200. doi: 10.4103/ijd.ijd_531_17, PMID: 29937554 PMC5996629

[20] Guarneri C Lotti J Fioranelli M Roccia MG Lotti T Guarneri F. Possible role of *Helicobacter pylori* in diseases of dermatological interest. J Biol Regul Homeost Agents. (2017) 31:57–77. PMID: 28702966

[21] Shamseer L Moher D Clarke M Ghersi D Liberati A Petticrew M . Preferred reporting items for systematic review and meta-analysis protocols (PRISMA-P) 2015: elaboration and explanation. BMJ. (2015) 349:g7647. doi: 10.1136/bmj.g7647, PMID: 25555855

[22] Lo CK-L Mertz D Loeb M. Newcastle–Ottawa Scale: comparing reviewers’ to authors’ assessments. BMC Med Res Methodol. (2014) 14:45. doi: 10.1186/1471-2288-14-45, PMID: 24690082 PMC4021422

[23] Higgins J Thompson SG Deeks JJ Altman DG. Measuring inconsistency in meta-analyses. BMJ. (2003) 327:557–60. doi: 10.1136/bmj.327.7414.557, PMID: 12958120 PMC192859

[24] Ribaldone DG Saracco G Pellicano R. Comment on *Helicobacter pylori* seroprevalence and the occurrence and severity of psoriasis. An Bras Dermatol. (2017) 92:584. doi: 10.1590/abd1806-4841.2017112, PMID: 28954122 PMC5595620

[25] Bardazzi F Magnano M Fiorini G Vaira D Odorici G Bertusi G . *Helicobacter pylori* infection in psoriatic patients during biological therapy. Ital J Dermatol Venereol. (2021) 156:570–4. doi: 10.23736/s2784-8671.19.06410-1, PMID: 32041937

[26] Mesquita PMD Diogo Filho A Jorge MT Berbert ALCV Mantese SADO Rodrigues JJ. Relationship of *Helicobacter pylori* seroprevalence with the occurrence and severity of psoriasis. An Bras Dermatol. (2017) 92:52–7. doi: 10.1590/abd1806-4841.20174880, PMID: 28225957 PMC5312179

[27] Campanati A Ganzetti G Martina E Giannoni M Gesuita R Bendia E . *Helicobacter pylori* infection in psoriasis: results of a clinical study and review of the literature. Int J Dermatol. (2015) 54:e109–14. doi: 10.1111/ijd.12798, PMID: 25808243

[28] Menter A Strober BE Kaplan DH Kivelevitch D Prater EF Stoff B . Joint AAD-NPF guidelines of care for the management and treatment of psoriasis with biologics. J Am Acad Dermatol. (2019) 80:1029–72. doi: 10.1016/j.jaad.2018.11.057, PMID: 30772098

[29] Boehncke W-H Schön MP. Psoriasis. Lancet. (2015) 386:983–94. doi: 10.1016/s0140-6736(14)61909-7 26025581

[30] Rendon A Schäkel K. Psoriasis pathogenesis and treatment. Int J Mol Sci. (2019) 20:1475. doi: 10.3390/ijms20061475, PMID: 30909615 PMC6471628

[31] Mezmale L Coelho LG Bordin D Leja M. Review: epidemiology of *Helicobacter pylori*. Helicobacter. (2020) 25:e12734. doi: 10.1111/hel.12734 32918344

[32] Li Y Choi H Leung K Jiang F Graham DY Leung WK. Global prevalence of *Helicobacter pylori* infection between 1980 and 2022: a systematic review and meta-analysis. Lancet Gastroenterol Hepatol. (2023) 8:553–64. doi: 10.1016/s2468-1253(23)00070-5, PMID: 37086739

[33] Balci DD Yalcin HP Ozer B Duran N Inan MU Yenin JZ. Identification of *Helicobacter pylori* by urea breath test and serology in the patients with psoriasis. J Eur Acad Dermatol Venereol. (2016) 30:50.26466833

[34] Ahmed AS Al-Najjar AH Alshalahi H Altowayan WM Elgharabawy RM. Clinical significance of *Helicobacter pylori* infection on psoriasis severity. J Interf Cytokine Res. (2021) 41:44–51. doi: 10.1089/jir.2020.0144, PMID: 33621131

[35] Yuan C He Y Xie K Feng L Gao S Cai L. Review of microbiota gut brain axis and innate immunity in inflammatory and infective diseases. Front Cell Infect Microbiol. (2023) 13:1282431. doi: 10.3389/fcimb.2023.1282431, PMID: 37868345 PMC10585369

[36] Wang L Cao ZM Zhang LL Dai XC Liu ZJ Zeng YX . *Helicobacter pylori* and autoimmune diseases: involving multiple systems. Front Immunol. (2022) 13:833424. doi: 10.3389/fimmu.2022.833424, PMID: 35222423 PMC8866759

[37] Kunovsky L Dite P Jabandziev P Dolina J Vaculova J Blaho M . *Helicobacter pylori* infection and other bacteria in pancreatic cancer and autoimmune pancreatitis. World J Gastrointest Oncol. (2021) 13:835–44. doi: 10.4251/wjgo.v13.i8.835, PMID: 34457189 PMC8371525

[38] Guarneri C Ceccarelli M Rinaldi L Cacopardo B Nunnari G Guarneri F. *Helicobacter pylori* and skin disorders: a comprehensive review of the available literature. Eur Rev Med Pharmacol Sci. (2020) 24:12267–87. doi: 10.26355/eurrev_202012_24019, PMID: 33336746

[39] Tshibangu-Kabamba E Yamaoka Y. *Helicobacter pylori* infection and antibiotic resistance—from biology to clinical implications. Nat Clin Pract Gastroenterol Hepatol. (2021) 18:613–29. doi: 10.1038/s41575-021-00449-x, PMID: 34002081

[40] Xiao-Hong S Yun-Sheng XU. Correlation between *Helicobacter pylori* infections and psoriasis vulgaris. Chin J Nosocomiol. (2013) 23:1100–1102.

[41] Azizzadeh M Nejad ZV Ghorbani R Pahlevan D. Relationship between *Helicobacter pylori* infection and psoriasis. Ann Saudi Med. (2014) 34:241–4. doi: 10.5144/0256-4947.2014.241, PMID: 25266185 PMC6074593

[42] Aihemaiti Z. Correlation between *Helicobacter pylori* infection and psoriasis vulgaris In: MA thesis. Ürümqi: Xinjiang Medical University (2023)

[43] Daudén E Vázquez-Carrasco MA Peñas PF Pajares JM García-Díez A. Association of *Helicobacter pylori* infection with psoriasis and lichen planus: prevalence and effect of eradication therapy. Arch Dermatol. (2000) 136:1275–6. doi: 10.1001/archderm.136.10.1275, PMID: 11030788

[44] Fabrizi G Carbone A Lippi ME Anti M Gasbarrini G. Lack of evidence of relationship between *Helicobacter pylori* infection and psoriasis in childhood. Arch Dermatol. (2001) 137:1529. PMID: 11708968

[45] Türkmen D Özcan H Kekilli E. Relation between psoriasis and *Helicobacter pylori*. Turk J Dermatol. (2011) 5:39–42. doi: 10.5152/tdd.2011.09

[46] Zhelezova G Yocheva L Tserovska L Mateev G Vassileva S. Prevalence of *Helicobacter pylori* seropositivity in patients with psoriasis. Probl Inf Parasit Dis. (2015) 43:1. doi: 10.27433/d.cnki.gxyku.2022.000803

[47] Qayoom S Ahmad QM. Psoriasis and *Helicobacter pylori*. Indian J Dermatol Venereol Leprol. (2003) 69:133–4. PMID: 17642857

[48] Minghua W Hengjin L. Psoriasis vulgaris and *Helicobacter pylori* infection. Med J Chin People’s Lib Army. (2008):600–602. doi: 10.3321/j.issn:0577-7402.2008.05.040

[49] Xie Y. Correlation analysis between psoriasis vulgaris and *Helicobacter pylori* infection. Chin J Clin Rat Drug Use. (2012) 5:112–3. doi: 10.15887/j.cnki.13-1389/r.2012.17.140

[50] Huang C Jiang Y Yao A Lv X Liu D. Correlation between *Helicobacter pylori* infection and unusual psoriasis. Chin J Dermatol. (2007) 40:645. doi: 10.3760/j.issn:0412-4030.2007.10.020

[51] Chen J Jiang Y. Detection of antibodies to *Helicobacter pylori* in patients with psoriasis vulgaris. Chin J Nosocomiol. (2012) 22:3748.

