# Supplementary figures and images for: Helicobacter pylori infection and its impact on psoriasis: a systematic review and meta-analysis
Source: Front Med (Lausanne). 2024 Dec 6;11:1500670. doi: 10.3389/fmed.2024.1500670 (PMC11659017; doi:10.3389/fmed.2024.1500670)

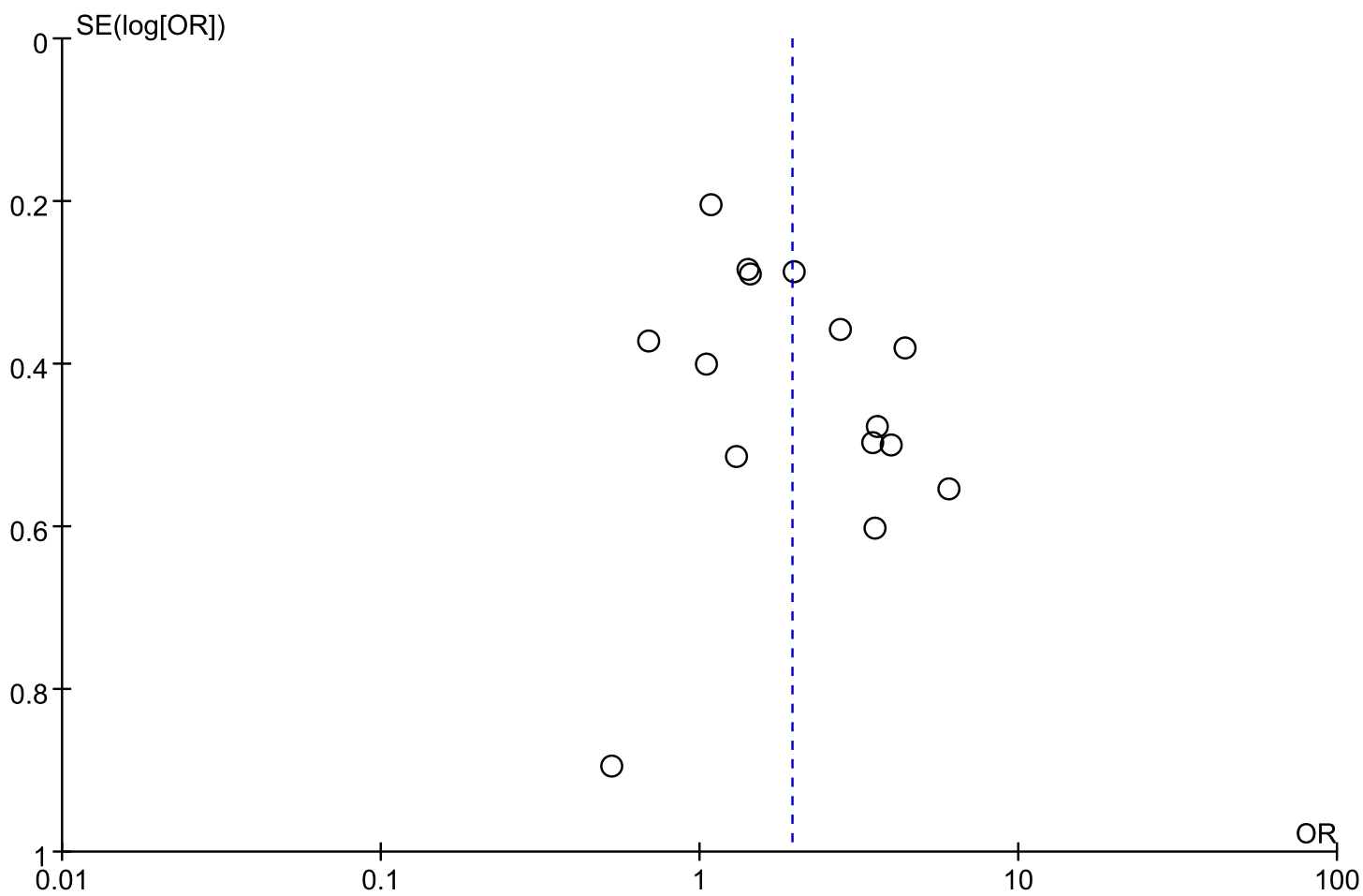

Supplement: Supplementary file 2 [file Image_1.pdf]
